# Study of the Effect of Temperature on the Production of Carrageenan-Based Buccal Films and Optimization of the Process Parameters

**DOI:** 10.3390/ph17121737

**Published:** 2024-12-22

**Authors:** Katalin Kristó, Anahita Sangestani, Alharith A. A. Hassan, Hala Rayya, Krisztián Pamlényi, András Kelemen, Ildikó Csóka

**Affiliations:** 1 Institute of Pharmaceutical Technology and Regulatory Affairs, University of Szeged, H-6720 Szeged, Hungarypamlenyi.krisztian@szte.hu (K.P.); csoka.ildiko@szte.hu (I.C.); 2 Department of Technical Informatics, Faculty of Science and Informatics, University of Szeged, H-6720 Szeged, Hungary; kelemen.andras.felix@szte.hu

**Keywords:** carrageenan, buccal film, mucoadhesion, dissolution, breaking hardness, deformation

## Abstract

Background/Objectives: Films in the mouth offer a promising alternative drug delivery system for oral administration, with several advantages over traditional oral formulations. Furthermore, their non-invasive nature and easy administration make them conducive to increasing patient compliance. The use of active agents in these films can further improve their drug delivery properties, making them an even more useful drug delivery system. Methods: In this research, carrageenan was used as a polymer, while glycerine was added as a plasticizer, furthermore, lidocaine hydrochloride and diclofenac sodium were used as the active agents. The prepared films were characterized by analytical techniques. Results: The results showed that glycerine reduced the mucoadhesivity and breaking hardness of the films and increasing the temperature made the films brittle. These results are also confirmed by the statistical analysis. Based on the FTIR results, glycerine can be used in films without structural changes. Conclusions: Based on the findings, films prepared from a solution with a concentration of 1.5% carrageenan and 1.5% glycerine at 70 °C are suitable as a drug delivery system for use on the buccal mucosa when combined with active agents. Carrageenan was successfully used as a carrier for two different types of active agents.

## 1. Introduction

Films in the mouth are thin, flexible, and transparent formulations that can be directly placed into the oral cavity and adhering to the mucosal surfaces. Films in the mouth offer several benefits as a drug delivery system, including enhancing the bioavailability of drugs, providing an excellent alternative for medication administration for children and the elderly, helping those with swallowing difficulties to ensure adequate medication intake, and minimizing side effects [1]. Furthermore, their non-invasive nature and easy administration make them conducive to increasing patient compliance [2,3,4]. The injection as a mode of application can be very inconvenient, and the evaluation of non-invasive routes is therefore very relevant. These films can also be used for the local delivery of drugs to treat various oral conditions such as mucositis, periodontitis, and halitosis. Furthermore, they can be used for the systemic delivery of drugs, such as anti-inflammatory agents, to treat systemic conditions [5]. The versatility and convenience of films in the mouth make them a promising alternative instead of traditional oral formulations. However, the development of films as a drug delivery system is still in its early stages, and further studies are needed to fully evaluate their potential as a safe and effective delivery system [6].

The oral epithelium is composed of several layers including mucus, stratum disten-dum, stratum filamentosum, stratum suprabasale, and stratum basale. The basement membrane is located beneath the epithelium, followed by the lamina propria, which serves as an underlying supportive connective tissue layer, and the submucosa [7]. The total surface area of the oral cavity is approximately 170 cm^2^, but only 50 cm^2^ of this [8] is considered pharmaceutically useful as the active pharmaceutical ingredient (API) can only be absorbed on the non-keratinized part [9,10]. The maxillary artery provides efficient circulation to this area, with a rate of 2.4 mL/min/cm^2^, which is better than the sublingual area and favorable for absorption [11,12].

The permeation sequence in the oral cavity regarding the mucosa is arranged as follows: the sublingual area is the most permeable, followed by the buccal region and then the soft palate region [13]. This variation in permeability makes the buccal region well suited for utilizing slow- and immediate release drug delivery systems, in contrast the sublingual area which is only a rapidly absorbed region [4]. A number of physicochemical properties should be possessed by an API to be a good candidate for delivery through buccal mucosa. Drugs with low molecular weight and slight water solubility are delivered effectively and faster through the buccal mucosa. In general, drug delivery through buccal mucosa decreases exponentially with molecular weights above 300 Da. Smaller molecules are mainly absorbed through passive transcellular diffusion or carrier-mediated transport (below 500 Da). A way to enhance the absorption of larger molecules is to regulate the “tight junctions” found within the tissue, which can be done using various excipients and mucoadhesive polymers [14]. The potential for a drug to be absorbed through the buccal mucosa depends on factors beyond its molecular weight, including its lipid solubility. Absorption of ionizable substances occurs transcellularly and depends on the pH, with the ionized form having worse penetration compared to the non-ionized one [15].

Mucoadhesion is the process by which mucosal substances and mucin are held together by an attractive bond for a long time. It is considered bioadhesion if both binders are biological. Mucin is a hydrophilic macromolecule and contains a large number of H atoms and OH groups, so it can form primary (covalent, ionic glycosides, ester bond) and secondary (H bond, van der Waals forces) bonds with biological structures [16,17]. Mucoadhesion involves several bonds, the main categories being adsorption theory, diffusion theory, wetting theory, fracture theory, and electrostatic theory [18]. Mucoadhesion force and mucoadhesion time are also important from the point of view of application, because if one of these two parameters is insufficient, absorption of the active agent cannot occur.

There are two main types of methods for producing polymer films, namely casting and melt extrusion [19]. Film casting, also known as the solvent evaporation-based method, is used more frequently. This method can be applied at room temperature or at higher temperatures to accelerate solvent evaporation, but it should be noted that room-temperature casting can also be used for heat-sensitive materials to form polymer films. The method has the advantage of being inexpensive and easy to implement [19,20,21]. Buccal films produced by the film casting method generally have sufficient stability, whereas films produced by the hot melt extrusion method may have chemical stability problems. There are active substances for which the use of a stabilizer is justified, such as for omeprazole [22]. The stability of the products after production is very important; therefore, it is necessary to protect the film products from light, moisture, oxygen, and mechanical effects [1].

Carrageenan is a type of sulphated polysaccharide that is extracted from red seaweed (Rhodophyceae). It is categorized into three grades based on the number of sulphate groups per repeat unit of polysaccharide: kappa (κ), iota (ι), and lambda (λ) with one, two, or three sulphate groups, respectively [23,24]. When carrageenan is dissolved in water, all grades produce a thermo-reversible sol–gel, which undergoes dispersion following random-coil formation in the sol stage. Despite being widely used in the food industry as a natural polymer, extensive research is currently being carried out in pharmaceutical applications, because it can not only be used as an excipient, but has also been shown to be capable of controlling viral infections, such as HPV, HSV, or SARS-CoV-2, bacterial infections, and even pathophysiological processes, such as hyperlipidemia. The carrageenans are highly safe, effective, biocompatible, biodegradable, and non-toxic [25]. Galactose sequences within the carrageenan chains twist in a double-helix fashion at low temperatures, and the sweet taste of galactose can mask the bitter taste of some drugs, avoiding the need for flavouring and sweetening agents [26]. Nowadays, carrageenan is mainly used as polymer matrix in oral extended-release tablets, as a novel extrusion aid for the production of pellets, and as a carrier or stabilizer in micro- and nanoparticles systems. Moreover, it has been used as a gelling agent [27] and viscosity-enhancing agent for controlled or prolonged drug release [28]. Furthermore, sulphate group in the carrageenan structure and the mucin present on the buccal mucosa can enhance the bioadhesive and mucoadhesive properties of the final formulation, respectively. Previous studies have also demonstrated an increase in the drug bioavailability of κ-carrageenan [29].

Some research groups have used carrageenan as a film-forming polymer for buccal application. Tejada et al. combined chitosan with λ and κ carrageenan for miconazole ap-plication and found that both λ and κ-carrageenan improved physical properties and mucoadhesion, although λ to a greater extent than κ [30]. Volod’ko et al. successfully produced multilayer buccal mucoadhesive films using chitosan and carrageenan and found that multilayer films are stronger than single-layer ones [31]. In the application of ibuprofen, paracetamol, and indomethacin in buccal films using glycerol and PEG as plasticizers, κ-carrageenan films can be promising drug delivery systems [32,33]. Boateng et al. produced films containing streptomycin and diclofenac sodium that can be used in wound healing, and based on structural analysis, they found that it can be used promisingly as dressing [34].

The objective of our work is to study and optimize the process parameters during the production of carrageenan buccal films, including the temperature, which, as far as we know, has not been investigated by other research groups. In order to produce films using casting technology, the first step is to produce a polymer solution, which in the case of κ-carrageenan, can be achieved by heating. Therefore, it is important to examine and optimize the temperature, too high a temperature can cause unfavorable properties in the film, as well as being economically unfavorable during production.

## 2. Results and Discussion

### 2.1. Film Thickness

**Figure 1:**
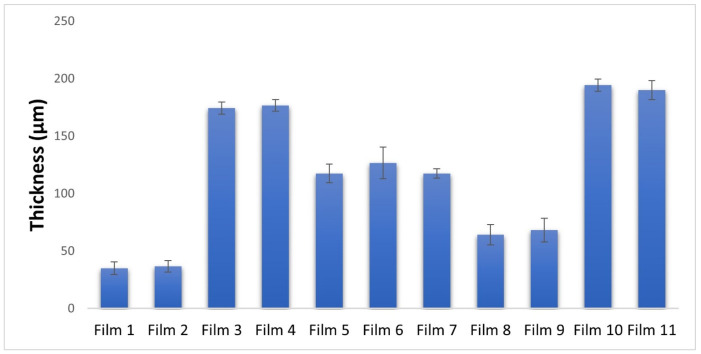
Graphical illustration of the thickness measurements of the polymer films.

The thickness of films can be greatly influenced by their composition, as evidenced by significant differences observed during the measurement process as presented graphically depicted in Figure 1. Interestingly, temperature was found to have no effect on the thickness variations of films with the same composition. Specifically, in films containing only polymer, there was a slight increase in thickness with increasing polymer concentration [35]. However, for films containing glycerine as a plasticizer, there was a significant increase in thickness that was directly proportional to the concentration of glycerine present. It can be seen that the highest values can be measured for Film 3, 4, 10, and 11 (Figure 1), but even these are well below 1 mm, where a unpleasant feeling may already occur. The explanation for this is that glycerine has hygroscopic properties, so the water content of the film will be higher, which is accompanied by an increase in the thickness of the film [36,37]. Films containing APIs were found to have thickness values similar to those without APIs, only being slightly higher in the case of diclofenac sodium. Overall, these findings highlight the crucial role that film composition can play in determining thickness, with certain components such as glycerine having a significant impact on this parameter. Having knowledge of and choosing the right thickness of polymer films is crucial for various reasons. The heavier weight of thicker films may impact the drug release rate and patient adherence, as a thick film applied to the buccal mucosa can generate an unpleasant feeling and a sense of foreign body, leading to discontinuation of use in some patients [37].

### 2.2. Breaking Hardness Test

**Figure 2:**
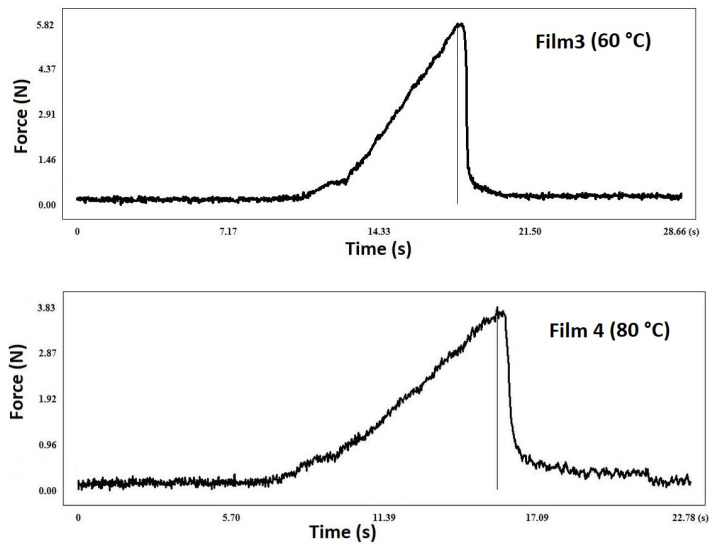
Deformation curve of films without API at 60 and 80 °C (Film 3 and 4).

**Figure 3:**
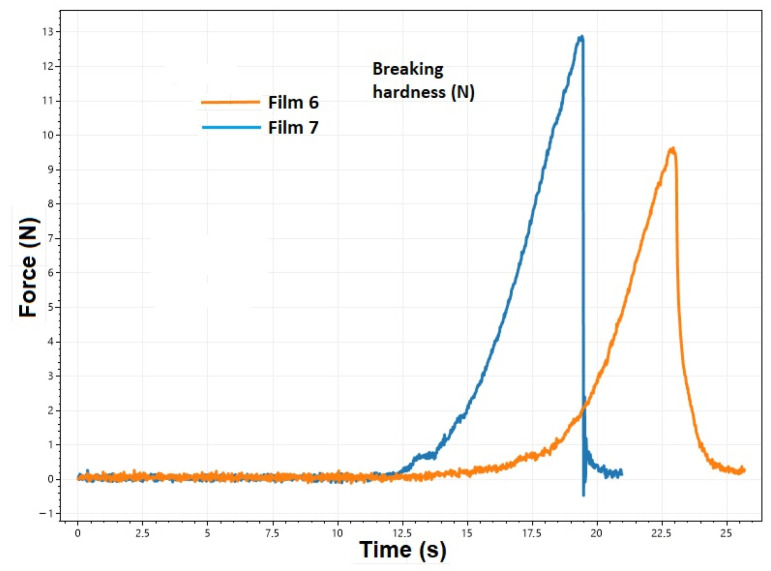
Deformation curve of films containing diclofenac sodium (Film 6) and lidocaine hydrochloride (Film 7).

The breaking hardness of a film is affected by the type and amount of polymer and plasticizer used, as well as the thickness of the film. Understanding the breaking hardness and plastic/elastic properties of the film is crucial for buccal mucoadhesive films. For the proper application of a film, it must possess both plastic and elastic properties. Elasticity is necessary for placing it on the buccal mucosa; therefore, the right amount of plasticizer is important for its storage and formulation [38,39]. Rigid, hard films show a steep curve as they are unable to deform much under force, causing them to break. In contrast, soft films deform under force and show a flatter curve, which also marks the end of the measurement [40]. Figure 2 shows the deformation curve of two films with the same composition produced at different temperatures. In the case of a film produced at 80 °C, higher elongation and lower breaking hardness can be observed, which suggests that the elastic properties have increased. This phenomenon was observed for all films with the same composition. The peak of the curve indicates the breaking hardness (Figure 2 and Figure 3). In the case of films containing the API, the elongation decreased and the breaking hardness increased (Figure 3), which indicates a lower elasticity; but at the same time, it can still be considered adequate from the point of view of applicability, because the fragility did not increase significantly.

**Figure 4:**
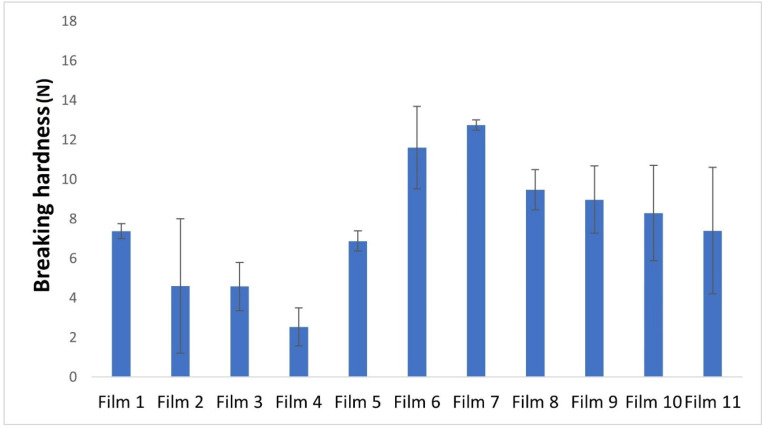
Graphical illustration of the breaking hardness of the polymer films.

Figure 4 shows the breaking hardness of different buccal film formulations.

It can be concluded that the concentration of carrageenan in films without glycerine has a direct correlation with breaking hardness. This is because higher amounts of polymer lead to the formation of more stable structures that are less prone to break.

However, there is another phenomenon with films containing glycerine. Glycerine serves to soften the film, but an increase in its concentration results in a decrease in breaking hardness. This is due to the interactions between glycerine and other film components, such as carrageenan. Glycerine typically forms hydrogen bonds and retains water, which increases the distance between polymer chains, making the film more susceptible to breakage [36]. As the water content in the film increases, so does the distance between the polymer chains, leading to a weaker backbone compared to films without glycerine.

Moreover, temperature also plays a role in determining the breaking hardness of the films, as mentioned above. Higher temperatures lead to lower breaking hardness, which may be related to film structure and integrity. This can be explained by the fact that evaporation is more intense at higher temperatures and more water can evaporate from the polymer film, which results in lower breaking hardness.

The addition of APIs such as diclofenac sodium and lidocaine hydrochloride increases the breaking hardness of the films. These findings can have important implications for the design and further optimization of buccal films for pharmaceutical applications.

Statistical analysis of buccal films plays a crucial role in characterizing their properties and determining their potential as a drug delivery system. In the case of breaking hardness (BH), the statistical analysis showed that the three-way interactions model well describes the effects of the three factors on the breaking hardness. X_2_X_3_ was ignored (1).

BH = 6.661 + 0.229C + **1.881X_1_** − **0.951X_2_** − **0.776X_3_** + 0.264X_1_X_2_ + 0.429X_1_X_3_ − 0.139X_1_X_2_X_3_ (1)

where C = curvature, X_1_ = carrageenan concentration, X_2_ = glycerine concentration, X_3_ = temperature

R^2^ = 0.99968, adj. R^2^ = 0.99744 and MS residual = 0.0136125

The significant factors are highlighted in bold. C in the equation indicates curvature and it was not significant in this equation.

**Figure 5:**
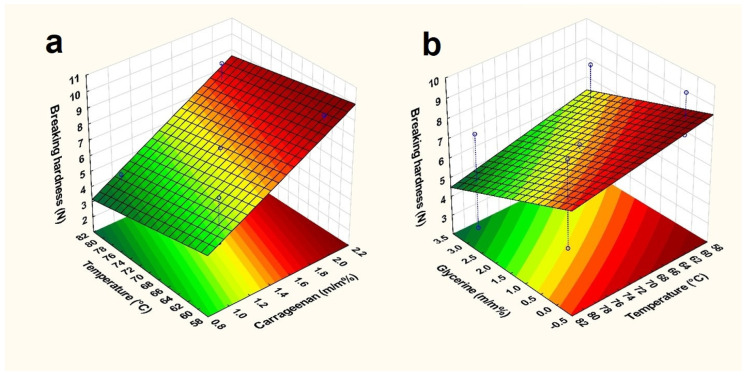
Response surface of breaking hardness ((**a**): temperature and carrageenan concentration; (**b**): glycerine concentration and temperature).

The addition of glycerine to films has the potential to enhance their elasticity, and flexibility. It can also be observed on the response surface that the presence of glycerine can also lead to a reduction in the breaking hardness of films (Figure 5b). Based on the statistical analysis, glycerine concentration is also a significant factor (x_2_). The other two factors, temperature (x_3_) and carrageenan concentration (x_1_), were also significant factors, which can be observed on the response surface (Figure 5) and in Equation (1), where the coefficient for carrageenan concentration has a + sign (+1.881), while the glycerine concentration and temperature also have a—sign (−0.951, −0.776). The coefficient with the + sign represents direct proportionality, so the higher the concentration of carrageenan, the higher the breaking hardness. And the—sign indicates inverse proportionality, so with the concentration of glycerine and the increase in temperature, the breaking hardness decreases.

On the other hand, the incorporation of a total carrageenan (x_1_) can have a positive effect on the breaking hardness of films, which is also statistically significant. This is because the polymer chains can create a cohesive and stable structure within the film, leading to stronger and more durable properties. The effect of the elevated temperature (x_3_), which was mentioned above, proved to be a significant factor based on Equation (1), and it can be observed on the response surface that the breaking hardness really decreases as the temperature increases.

### 2.3. In Vitro Mucoadhesion Test

**Figure 6:**
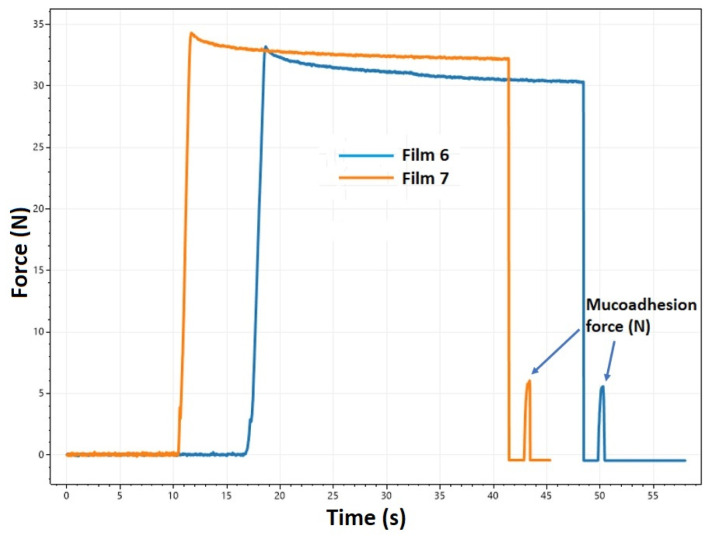
Mucoadhesion force measurements of films containing diclofenac-sodium (Film 6) and lidocaine hydrochloride (Film 7).

Determining the mucoadhesive force is crucial for buccal and other mucoadhesive dosage forms. This parameter represents the force necessary to separate the mucoadhesive film from mucin and buccal mucosa (Figure 6) [41].

**Figure 7:**
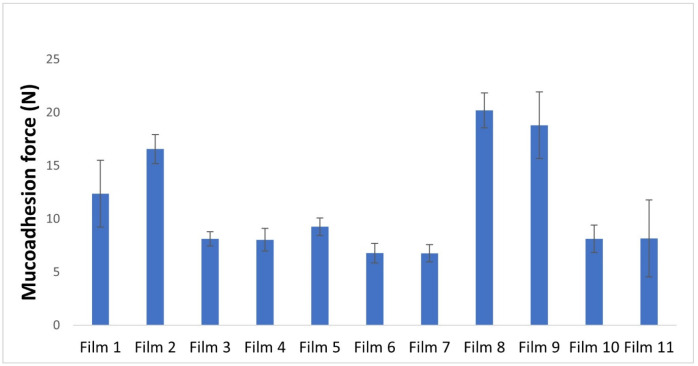
Graphical illustration of the mucoadhesion force of the polymer films.

The results of the study on the mucoadhesion of the prepared films are graphically depicted in Figure 7 for easier comparison. The films without APIs showed higher mucoadhesion force values; on the other hand, the mucoadhesion force of the diclofenac sodium and lidocaine hydrochloride-containing samples was significantly lower, with an average value of 6.78 N and 6.76 N. This reduction in mucoadhesion force of the polymer films could be due to the interaction between the sulphate groups of carrageenan, diclofenac sodium, and lidocaine hydrochloride molecules, resulting in fewer binding groups available for mucin binding. However, these samples still exhibited moderate mucoadhesion, which is sufficient for buccal drug delivery [36]. Increasing the total amount of polymers increased mucoadhesion due to the higher number of free-binding groups available for mucin binding in the system [42].

**Figure 8:**
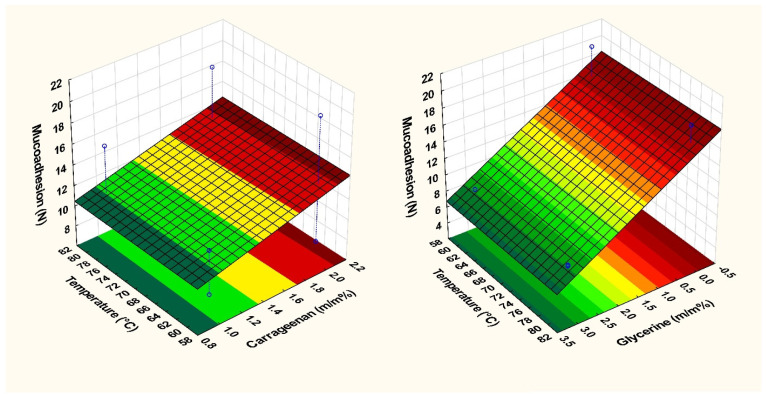
Response surface of mucoadhesion force.

The amount of glycerine used in the films was found to influence the mucoadhesion force. The data indicate that the presence of glycerine in the system was a statistically significant factor (2). This conclusion is backed up by the results obtained from Films 3 and 10, as well as Films 4 and 11, which had different concentrations of carrageenan and the same amount of glycerine but showed no significant difference in mucoadhesion. It is clearly visible on the response surface (Figure 8) that increasing the amount of glycerine resulted in a decrease in mucoadhesion force, possibly due to the formation of hydrogen bonds between glycerine and the film-forming polymers, as well as a lower number of free chains available in the polymer for mucin binding. It has been observed that temperature did not have a significant impact on mucoadhesion. In the case of films containing APIs (Film 6 and Film 7), a small decrease can be observed, but this does not affect applicability, but must be taken into consideration when planning the API content. For film containing diclofenac sodium, the ex vivo mucoadhesion force was 2.06 ± 0.58 N, while for lidocaine hydrochloride, it was 2.43 ± 0.13. Compared with the in vitro results, it can be seen that the ex vivo results, are approx. half of the in vivo values, but the results are not comparable due to the reduction of the applied pressure. Compared with the literature data, e.g., mucoadhesion forces of 1.49 N and 0.378 N were measured for 3D-printed films [43]. Baus et al. different polymers (hydroxyethyl cellulose, carboxymethyl cellulose, carbopol, polycarbophil, alginate, and xanthan gum) were tested and lower results were obtained, the maximum measured value was 1.5 N [44]. In the case of the films examined in this study, a higher mucoadhesion force can be measured compared to the literature data.

Indeed, as seen in Equation (1), temperature had a significant effect on breaking hardness. It has a negative sign; therefore, the higher the temperature, the lower the breaking hardness of the films. This was not observed in the case of mucoadhesion force, where temperature was the smallest factor, and it practically did not affect mucoadhesion force (Equation (2)). This can also be observed on the response surfaces, where mucoadhesion force did not change with temperature (Figure 8). The Equation (2) describes how mucoadhesive strength changes with temperature and glycerine concentration. This is the equation of the response surface shown in Figure 8 The factors x_1_ (temperature) and x_2_ (glycerine concentration) are included in the equation, the multiplier of which is the corresponding coefficient, which provides information about the effect of the given factor. In this case, e.g., the coefficient of x_2_ (glycerine concentration) is −4.310, where the—sign indicates that there is an inverse proportionality between the amounts, so the higher the concentration of glycerine, the lower the mucoadhesion. In this case, it was also statistically significant based on the results calculated by the Tibco statistical software.

In the case of mucoadhesion force (MS), the statistical analysis showed that the three-way interactions model well describes the effects of the three factors on the mucoadhesion force. In this case the X_3_ was ignored (2).

MS = 12.428 − 3.158C + 1.403X_1_ − **4.310X_2_** − 1.365X_1_X_2_ − 0.558X_1_X_3_ − 0.230X_2_X_3_ + 0.585X_1_X_2_X_3_ (2)

where C = curvature, X_1_ = carrageenan concentration, X_2_ =glycerine concentration, X_3_ = temperature

R^2^ = 0.99805, adj. R^2^ = 0.9844, and MS residual = 0.37845

The significant factors are highlighted in bold. The C in the equation indicates curvature and it is not significant in this equation.

Overall, the addition of more carrageenan to the system can enhance mucoadhesion by providing more free-binding groups for mucin to bind to but it was not significant. However, the presence of glycerine in the films was found to have a significant factor. In contrast, it can be observed that temperature did not significantly affect mucoadhesion in our experiments.

### 2.4. Fourier-Transform Infrared Spectroscopy (FTIR) Measurement

**Figure 9:**
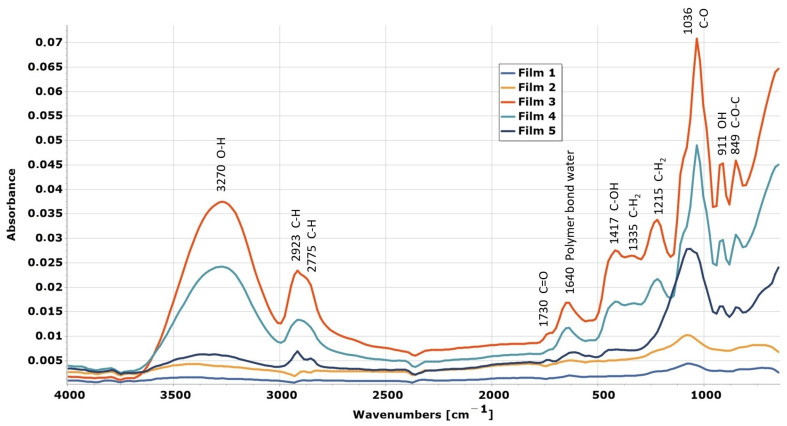
FTIR spectra of polymer films.

During our work, we recorded the FTIR spectrum of the films and then assigned the peaks that appeared. Based on the literature data [45], we found that the peaks that appear on the spectrum are mainly those arising from glycerine, but peaks also appeared at 1640 cm^−1^ and 849 cm^−1^, which may originate from carrageenan. The latter can be C-O-C or C-O-S axial secondary sulphate on C4 of galactose [46]. The spectra shown in Figure 9 show the FTIR spectra of films made with 1% and 1.5% polymer concentration. It can be seen that in the case of samples with the same composition but produced at different temperatures, the peaks are located in the same place, no new peaks appeared, and none of the peaks shifted. Therefore, it can be concluded that the temperature does not cause changes in the structure. The peaks of glycerine and carrageenan are visible on the spectrum at different concentrations of glycerine. Overall, glycerine can be safely and effectively used as a plasticizer in carrageenan-based buccal mucoadhesive films.

**Figure 10:**
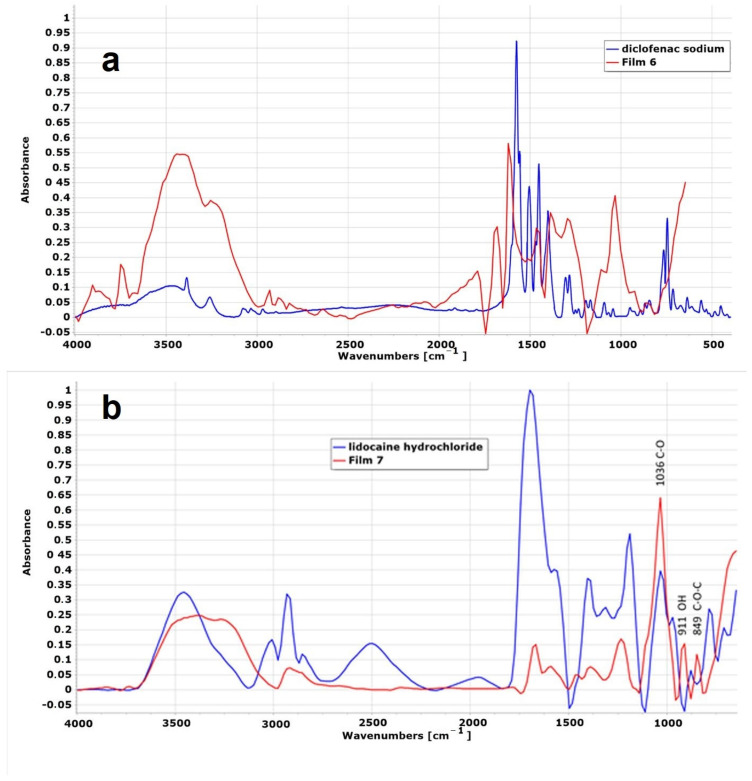
FTIR spectra of polymer films containing active agents: (**a**) diclofenac sodium; (**b**) lidocaine hydrochloride.

In the case of Film 7, some peaks characteristic of lidocaine hydrochloride can be identified (Figure 10), namely 2930, 1667, and 1034 cm^−1^. Peak 1667 cm^−1^ is a strong peak, which can be the carbonyl group stretching of the amide group [47]. In the additional region, peaks characteristic of κ-carrageenan were identified, such as 1034, 911, and 849 cm^−1^.

In the case of diclofenac sodium, there are greater differences, and practically, the characteristic peaks of diclofenac are not observed in the film containing diclofenac sodium (Figure 10). This suggests that chemical reactions took place between the polymer and the active ingredient. This is also supported by the fact that, based on our observations, the solution used to prepare the diclofenac films was opalescent, as was the dry film containing diclofenac. In contrast, in the case of lidocaine, both the solution and the film were completely transparent.

### 2.5. Loss on Drying

Measuring the loss on drying of buccal films is crucial for multiple reasons. Firstly, the amount of moisture in the film can affect its stability and shelf-life. An excessive amount of moisture can lead to microbial growth, degradation, and reduced effectiveness. Conversely, if the film is too dry, it can become brittle, lose flexibility, and break during the application, and not adhere well to the buccal mucosa [48]. Secondly, the moisture content can impact the drug release profile from the buccal film. The rate and extent of drug release can be affected by the amount of water in the film. Therefore, measuring the moisture content can ensure consistent drug delivery from batch to batch.

**Figure 11:**
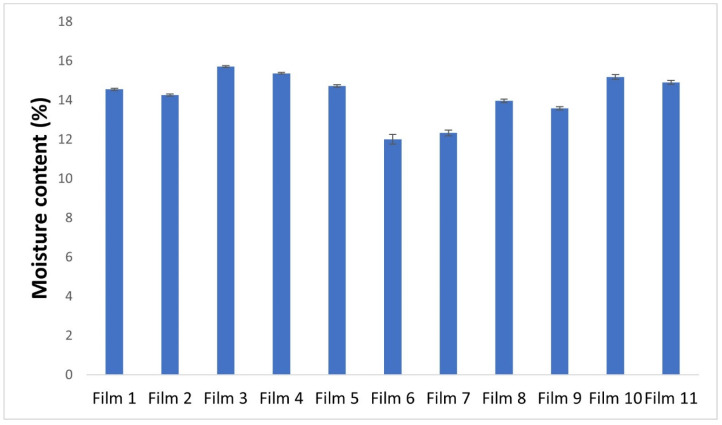
Graphical illustration of the loss on drying of the polymer films.

The results of the study on the loss on drying measurement of the prepared films are illustrated graphically in Figure 11. All results were between 12 and 14%. This hydrophilic polymer is capable of absorbing water, leading to an increase in water content in the film. However, at higher concentrations, kappa-carrageenan can form a gel network that entraps water, thereby potentially reducing the water content in the film. By increasing the concentration of carrageenan in our experiments, a decrease in moisture content was observed. Films containing glycerine have been found to have a higher moisture content than films without glycerine (Film 3, Film 4, Film 10, and Film 11). This can be attributed to the hygroscopic nature of glycerine, which can attract and bind water molecules. The hydroxyl groups present in glycerine molecules have the ability to form hydrogen bonds with water molecules, allowing for increased water absorption from the surrounding environment when added to a polymer matrix. The addition of glycerine to a film-forming system can thus lead to an increase in water content. In summary, measuring the loss on drying of buccal films is a critical quality control parameter that ensures the safety, efficacy, and stability of the product. The moisture content of films containing 3% glycerine and 2% polymer is around 15%, which means that the polymer film contains 51% glycerine and 34% polymer. The same film cast from a solution containing 1% glycerine results in a film containing 29% glycerine and 58% polymer, since the moisture content here was lower, at 13%. A solution of 1% glycerine and 1% polymer resulted in a film containing 43.5% glycerine and 43.5% polymer.

It can also be observed that it was the smallest in the case of samples containing APIs (12.01 and 12.33%) (Film 6 and Film 7), which can be explained by the fact that these APIs bind water less than carrageenan.

### 2.6. Dissolution Test

**Figure 12:**
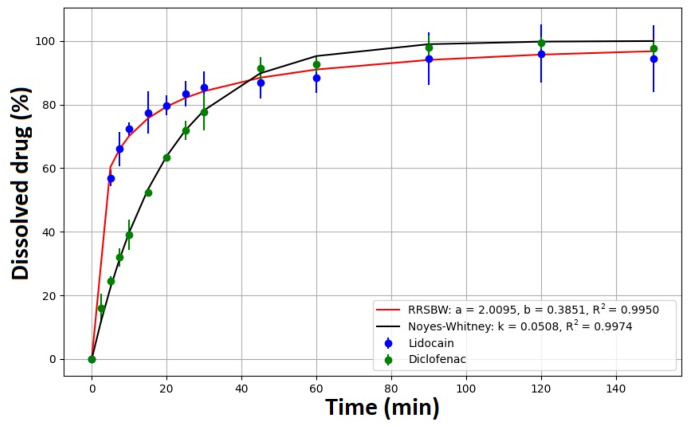
Dissolution curve of films containing diclofenac sodium (Film 6) and lidocaine hydrochloride (Film 7).

Dissolution tests were also performed on the films that contained the API, which are critical for their application. The API content was 4.75 mg in the case of the sample containing lidocaine hydrochloride, while 4.46 mg of diclofenac sodium in the sample used for the dissolution test. Through these tests, we recognized that the complete quantity of the API dissolves from every film composition. Figure 12 displays the dissolution profile for the mucoadhesive films containing diclofenac sodium and lidocaine hydrochloride. Lidocaine hydrochloride film releases the API at a faster rate than diclofenac sodium film. After 30 min, more than 78% of diclofenac sodium is released from the film, and over 92% is released within 45 min. The entire amount is released after 120 min, as the cumulative amount of released diclofenac sodium is relatively constant at the next point. Lidocaine hydrochloride shows a faster release than diclofenac sodium within the first 20 min, with a steep increase in the release curve. Specifically, 77.7% of lidocaine hydrochloride is released within this time frame, while for diclofenac sodium, it is only 63.68%. However, after 20 min, the release of lidocaine hydrochloride slows down and reaches 83% within 45 min. The entire amount is eventually released after 120 min, as the cumulative amount of released lidocaine hydrochloride remains relatively constant. Based on the results of this experiment, we can classify these films as relatively fast-release films. This is because the films containing lidocaine hydrochloride release over 50% of the API within 5 min, and films containing diclofenac sodium release over 50% of the API within 20 min.

Depending on the shape of the dissolution curve, various mathematical models can be used to describe the dissolution process, e.g.: zero-order, first-order, Noyes–Whitney, Higuchi, Hixson–Crowell, Korsmeyer–Peppas, Langenbucher, Rosin–Rammler–Sperling–Benett–Weibull (RRSBW) [49]. In this study, the fit of the above-mentioned models was examined, based on which it can be concluded that the Noyes–Whitney (lidocaine hydrochloride) (R^2^ 0.9974) and RRSBW (diclofenac sodium) (R^2^ 0.9950) models are suitable for fitting the dissolution curves (Figure 11). The Noyes–Whitney model can be derived from Fick’s first law. Fick’s first law describes the flux of particles through a medium due to diffusion. It states that the flux, J, is proportional to the concentration gradient, ∇C(r,t). Mathematically, it is expressed as Equation (3):

(3) J=−D∇C(r,t)

where

J is the diffusion flux (amount of substance per unit area per unit time);

D is the diffusion coefficient (a constant that depends on the nature of the diffusing substance and the medium);

∇C is the concentration gradient (the rate of change of concentration with respect to distance).

The negative sign indicates that diffusion occurs in the direction of decreasing concentration, so particles move from regions of higher concentration to regions of lower concentration. In a one dimensional case, Equation (3) can be rewritten to the following form (4):

(4) $$\frac{dC}{dt}=\frac{DA}{h}\left(C_{s}-C\right)$$

where

Cs = saturation solubility of drug;

C = concentration of drug in bulk solution;

D = diffusion coefficient of the drug;

A = surface area of drug;

h = thickness of stagnant layer.

Equation (4) is ordinary first-order differential equation, when integrated, yields Equation (5):

(5) $$M\left(t\right)=M_{0}(1-e^{-kt})$$

where

$k=\frac{DA}{h}$ is a dissolution rate coefficient;

M(t) is the amount of API dissolved as a function of time t;

M_0_ is the maximum amount of API dissolved.

In the case of diclofenac sodium, the RRSBW model fitted best. The RRSBW distribution is generally described by Equation (6):

(6) $$M\left(t\right)=M_{0}(1-e^{-\frac{{\left(t-T\right)}^{\beta }}{\alpha }})$$

where

T is the lag time;

α is a scale parameter that describes the time dependence;

β describes the shape of the curve.

For β=1 and T = 0, the shape of the curve corresponds exactly to the Noyes–Whitney equation with constant $k=\frac{1}{\alpha }$. The difference in the dissolution kinetics of the two APIs can be explained by the fact that lidocaine is a cationic API and dissolves easily in the carrageenan solution, while the anionic diclofenac takes more time to dissolve, which was also observed during production. In order for the API to dissolve, it must first dissolve, which is why lidocaine dissolves faster and diclofenac dissolves more slowly. In the case of diclofenac sodium, the diffusion theory can clearly be applied, while in the case of lidocaine hydrochloride, it can be seen that the RRSBW model is identical to the Noyes–Whitney equation in addition to the above-mentioned parameters, so the diffusion theory can also apply in this case.

## 3. Materials and Methods

### 3.1. Materials

Kappa-carrageenan (Gelcarin GP911 NF, FMC Biopolymer, 1735 Market Street Philadelphia Pennsylvania 19103, USA) was utilized as the foundation of the film. Glycerine (Merck KGaA, 64271 Darmstadt, Germany) was used as a plasticizer in the formulation. Lidocaine hydrochloride (Ph. Eur.) and diclofenac sodium (Ph. Eur.) were the API in the polymer films. Mucin (Sigma-Aldrich, China, mucin from porcine stomach Type II) (10 *w*/*w*%) was used in the evaluation of the mucoadhesion process and in determining the mucoadhesive force. Phosphate buffer (pH = 6.82) was employed as dissolution media in the dissolution test and double distilled water (Ph. Eur.) was used in the preparation of different solutions.

### 3.2. Methods

#### 3.2.1. Preparation and Formulation of Polymer Films

In the first step, films without API were prepared and aimed to determine the optimal temperature and concentration of carrageenan and glycerine for the addition of the API. The casting method was carried out at three different temperatures. Κ-carrageenan was added to distilled water in small portions while stirring until it dissolved completely. Then, the resulting polymer solution was mixed with glycerine and poured into the plastic dishes, allowing it to evaporate at room temperature. In the next step, films containing diclofenac sodium and lidocaine hydrochloride were produced using the same casting method at 70 °C with solvent evaporation.

The concentration of the polymer solution used to produce the films, and API amount, is shown in Table 1. The applicability of two APIs, cationic lidocaine hydrochloride and anionic diclofenac sodium, was investigated in carrageenan. During formulation, it can be seen that lidocaine hydrochloride dissolves quickly, while diclofenac sodium took longer. After drying the films, it can be observed that the transparency of the films containing lidocaine hydrochloride was higher than the films containing diclofenac sodium. The solubility of diclofenac sodium in the literature is 20.4 mg/mL [50] and 19.4 mg/mL [51], while that of lidocaine hydrochloride is 54 mg/mL in water at 25 °C, which affects the difference. To ensure the desired amount of API in each film, 2 × 2 cm squares were cut from the finished films.

#### 3.2.2. Film Thickness Measurement

To measure the thickness of polymer films, a micrometer screw gauge with a measurement range of 0–25 mm and a resolution of 0.001 mm (Mitutoyo Co. Ltd., Kawasaki, Japan) was utilized. Six parallel measurements were carried out and the average values and standard deviations were calculated.

#### 3.2.3. Breaking Hardness Measurement

The behavior of plastic and elastic properties of films can be determined by analyzing their deformation curves. The breaking hardness of a film is measured in Newtons when it reaches its breaking hardness point and breaks. The breaking hardness was measured using a texture analyzer developed at our institute [28]. The device has two parts: a circular metal sample holder that secures the film and a rounded shape that can apply pressure to the film, causing it to break in most cases. The force–time curve is continuously monitored during the measurement, and the properties of the recorded curve allow us to infer the plastic/elastic nature of the film [29].

The device consisted of a fixed disc (20 mm in diameter) and a movable sample holder that could accommodate different sample holders depending on the investigation. The force (in the range of 0–200 N), moving speed, and time could be recorded. The hardness test was performed using a needle-like probe (3 mm diameter) that was moved downward at a constant speed (2 mm/s). The film was fixed at the bottom of the equipment, and the probe was passed through the film. The output was 0–5 V, and the sampling rate was 50 Hz. The time and force were detected during the investigation, and the test was terminated when the film was broken. During our research, six measurements were performed in parallel, and the average value was calculated. In addition, standard deviations were calculated.

#### 3.2.4. In Vitro and Ex Vivo Mucoadhesion Test

To measure the in vitro mucoadhesive force of each film composition, a device developed by the institute was used, which also made it possible to measure the mucoadhesion force. For each measurement, the film was placed on the flat surface of a rod-like probe with a diameter of 9 mm, which had a large area and a double-sided adhesive attached to it. Then, 40 µL of a freshly prepared mucin solution (10% *w*/*w*) was spread on a 35 mm circular disc assembled at the bottom of the equipment. The rod-like probe was moved down towards the bottom disc, and a 30 ± 0.1 N force was applied for 30 s to bring the film into contact with the mucin. Following that, the probe was moved upwards, and the force required to detach the film from mucin was presented in the form of a peak in the force–time curve. The detachment force of the film corresponds to the displayed peak maximum [29]. The test was repeated at least 6 times, and the means and standard deviations were calculated. Ex vivo tests were performed for films containing active ingredients. A Leica CM1950 cryostat (Leica Biosystems GmbH, Wetzlar, Germany) was used to prepare 15 μm thick cross-sections of porcine buccal mucosa. In this case, the compressive force had to be reduced to 10 N, but the other parameters were constant.

#### 3.2.5. Fourier-Transform Infrared Spectroscopy (FTIR) Measurement

The FTIR spectra of the excipients and polymer films were carefully analyzed using advanced equipment. Specifically, an Avatar 330 FTIR apparatus (Thermo Fisher Scientific Inc. Waltham, MA, USA) was utilized, along with coupled Zn/Se horizontal attenuated total reflectance (HATR) equipment. The films were delicately placed on a clean crystal of the apparatus, and scanned for absorbance in the wavelength range of 600 to 4000 cm^−1^. The spectra were obtained from 128 scans at a high spectral resolution of 4 cm^−1^, while also accounting for the effects of CO_2_ and H_2_O through careful correction techniques. SpectraGryph (version 1.2.15.; Dr. Friedrich Menges Software, Entwicklung, Obersdorf, Germany) was used in the evaluation of the results.

#### 3.2.6. Loss on Drying Measurement

In this study, a halogen moisture analyzer (MAC 50/NH, RADWAG Wagi Elektroniczne, Radom, Poland) was utilized for measuring the loss on drying of the buccal film samples. The analyzer operates by placing at least 0.1 g of the sample inside it and then using a heating element, such as a halogen lamp, to evaporate the moisture in the sample. The measurements were based on a thermogravimetric principle, as the mass loss upon drying was recorded and expressed as a percentage of the original mass. To ensure precision, the test was repeated three times, and the mean value was calculated.

#### 3.2.7. Dissolution Test

In this study, the Multiposition Digital Hotplate Stirrer (VELP, Malaysia) was used to perform dissolution tests on all compositions containing API. The dissolution medium used was 50 mL of pH 6.8 phosphate buffer solution at 37 °C with a mixing speed of 100 rpm. Twelve measurements were conducted, and samples were obtained manually by pipette (5 mL) at specific time intervals (2.5, 5, 7.5, 10, 15, 20, 30, 45, 60, 90, 120, 150 min). The samples were analyzed using a Genesys 10S UV–VIS spectrophotometer at a wavelength of 263 nm for lidocaine hydrochloride and 276 nm for diclofenac sodium after calibration.

#### 3.2.8. Statistical Analysis

The data collected in this study were analyzed using the factorial ANOVA method, using Tibco Statistica v13.4.0.14 (Statsoft Inc., Tulsa, OK, USA) software. Two-level full factorial design with one central point was adopted as a design of experiment in this study. Equations were developed to describe the relationship between the three factors (x_1_-total polymer concentration, x_2_-concentration of glycerine and x_3_-temperature) and the two optimization parameters (y_1_-breaking hardness, y_2_-mucoadhesion force). The minimum, maximum, and central points of the factors are shown in Table 2.

## 4. Conclusions

Pharmaceutical companies are actively developing new methods, processes, and products, and one of the potential products under development is oral mucoadhesive systems. These systems offer several advantages, such as the ability to address swallowing difficulties. The primary objective of this study was to create buccal mucoadhesive polymer films and evaluate their physical and physical–chemical properties. The results showed that the addition of glycerine reduced the mucoadhesivity and breaking hardness of the films, and increasing the temperature made the films have lower breaking hardness and higher elongation, which indicates an increase in elasticity. These results are also confirmed by the results of the statistical analysis, because in the case of breaking hardness, all factors, i.e., carrageenan concentration, glycerine concentration, and temperature were also significant factors. In the case of mucoadhesion, the glycerol concentration proved to be a significant factor. At the same time, the preparation time is very long at 60 °C, so 70 °C was chosen, where the films can still be applied. In this study, the temperature used in the production of carrageenan films was investigated and optimized for the first time. Based on the FTIR results, it can be concluded that glycerine can be used in carrageenan films without any structural changes. Based on the optimization, films containing 1.5% carrageenan and 1.5% glycerine concentrations when produced at 70 °C are considered suitable for use as a drug delivery system on the buccal mucosa when combined with APIs. The addition of APIs increased the breaking hardness of films, while the APIs completely dissolved from all the films and the diffusion theory could be applied. Thus, carrageenan was successfully used as a carrier for two different types of APIs taking into account possible interactions.

## Figures and Tables

**Table 1:** The concentration of the polymer solution used to produce the films.

	Carrageenan (*w*/*w*%)	Glycerine (*w*/*w*%)	Temperature (°C)	Diclofenac Sodium (0.5 *w*/*w*%)	Lidocaine Hydrochloride (0.5 *w*/*w*%)
Film 1	1	0	60	-	-
Film 2	1	0	80	-	-
Film 3	1	3	60	-	-
Film 4	1	3	80	-	-
Film 5	1.5	1.5	70	-	-
Film 6	1.5	1.5	70	+	-
Film 7	1.5	1.5	70	-	+
Film 8	2	0	60	-	-
Film 9	2	0	80	-	-
Film 10	2	3	60	-	-
Film 11	2	3	80	-	-

**Table 2:** Levels of factors in the full factorial design.

Factors	Minimum Level	Maximum Level	Central Point
Total polymer concentrations (x_1_)	1%	2%	1.5%
Concentration of glycerine (x_2_)	0%	3%	1.5%
Temperature (x_3_)	60 °C	80 °C	70 °C

## Data Availability

The data presented in this study are available on request from the corresponding author.
